# PI3Kα Inhibitors That Inhibit Metastasis

**DOI:** 10.18632/oncotarget.166

**Published:** 2010-09-11

**Authors:** Oleg Schmidt-Kittler, Jiuxiang Zhu, Jian Yang, Guosheng Liu, William Hendricks, Christoph Lengauer, Sandra B. Gabelli, Kenneth W. Kinzler, Bert Vogelstein, David L. Huso, Shibin Zhou

**Affiliations:** ^1^The Ludwig Center for Cancer Genetics and Therapeutics and Howard Hughes Medical Institute, Johns Hopkins Kimmel Cancer Center, Baltimore, MD 21231, USA; ^2^The Sidney Kimmel Comprehensive Cancer Center and Department of Molecular and Comparative Pathobiology, Johns Hopkins University School of Medicine, Baltimore, MD 21205, USA; ^3^Department of Biophysics and Biophysical Chemistry, The Johns Hopkins University School of Medicine, Baltimore, MD 21205, USA

**Keywords:** PI3K, metastasis, small molecule inhibitor

## Abstract

Previous genetic analyses have suggested that mutations of the genes encoding PI3Kα facilitate invasion and metastasis but have less effect on primary tumor growth. These findings have major implications for therapeutics but have not been factored into pre-clinical drug development designs. Here we show that the inhibition of PI3Kα by newly designed small molecule inhibitors prevented metastasis formation in mice but had much less effect on the growth of subcutaneous xenografts or primary intra-abdominal tumors. These data support the idea that PI3Kα plays an important role in the metastatic process and suggest a more informed strategy for selecting drugs worthy of further development for clinical application.

## INTRODUCTION

The phosphatidylinositol-3-kinases (PI3K) class 1A family is a critical regulator of growth, motility and survival functions [1]. PI3K class 1A members transmit signals from a variety of growth factor receptors, creating phosphatidylinositol 3,4,5-trisphosphate (PIP3). PIP3 serves as activator and membrane docking station for PDK1 and Akt proteins, thereby initiating a kinase cascade that connects the cell membrane receptors with nuclear transcription factors. In light of its central role in numerous cellular processes, the phosphorylation of phosphatidylinositol 4,5-bisphosphate (PIP2) is tightly regulated, with PTEN reversing the phosphorylation of PIP2 catalyzed by PI3K Class 1A family members.

The alpha isoform of PI3K (PI3Kα) is one of the most frequently mutated kinases in solid tumors [2, 3]. Structural insights from the study of normal and mutated forms of PI3Kα suggest a release of inhibition by its regulating subunit p85 as well as altered membrane binding as causes for the observed biochemical over activity of the mutants [4-8]. The mutational activation of PI3Kα permits cell survival in culture when growth factors are limiting [6, 8].

PI3Kα is a heterodimer of a catalytic subunit encoded by *PIK3CA* and one of several regulatory subunits [9]. The high frequency of *PIK3CA* mutations in human tumors, the localization of mutations to particular “hotspot” regions, and the enhanced enzymatic activity of the mutant gene's products have made PI3Kα a preferred target for drug development. Indeed, *PI3KCA* is one of the two most highly mutated oncogenes ever discovered (the other being *KRAS*). For these reasons, many academic and industrial groups are attempting to develop inhibitors of this enzyme [10-21]. There are currently nine PI3K inhibitors in clinical trials, none of which are particularly specific for PI3Kα. These inhibitors can be classified into six core structures. PX-866 and SF1126 are analogues of wortmannin and LY294002, respectively, with improved pharmacokinetic properties [13, 22]. NVP-BEZ235, PF-04691502, BGT226 and XL765 are novel compounds with a broader and more potent inhibition profile that target PI3Ks as well as mTOR [23-25]. GDC-0941, XL147 and NVP-BKM120 are potent pan PI3K class 1A inhibitors lacking mTOR inhibition [26, 27].

Metastases, rather than the primary tumors themselves, are the cause of death in the vast majority of cancer patients. Interestingly, cancer cell lines in which the spontaneously mutated PI3Kα is genetically disrupted still grow as xenografts in nude mice, though their metastatic ability is severely compromised [8]. Similarly, cell lines in which Akt1 and Akt2 are genetically disrupted form xenografts at their primary sites but do not metastasize as well as their parental lines [28]. Overexpression of Akt1 has also been shown to enhance metastasis in rodent models and Akt2 knockdown inhibited such metastasis [29]. Clinical evidence of the importance of the PI3K/Akt pathway in metastasis development has also been documented [30, 31].

The purposes of the current study were (i) to introduce a novel set of PI3Kα specific inhibitor analogs based on an imidazopyridine core, and (ii) to test the most promising of these compounds for their in vivo activities against primary and metastatic tumors.

## RESULTS

### Synthesis and structure–activity relationship (SAR) studies of imidazopyridine-based J-series compounds

We synthesized 121 compounds based on N'- [(1E)-(6-Bromoimidazo [1,2-a]pyridin-3-yl)methylene]-N,2-dimethyl-5-nitrobenzenesulfonohydrazide hydrochloride (J27), a potent PI3Kα inhibitor reported previously [14]. Cyclization of 5-bromo-2-aminopyridine with chloroacetaldehyde gave imidazopyridine, which was formylated and further reacted with various hydrazines to provide the key intermediate compound 3. Acylation or sulfonylation of compound 3 generated the desired compounds (Supplementary scheme 1). Each of the compounds was tested for selective biochemical inhibition of PI3K isoforms in vitro. For this purpose, a baculovirus-based expression system was used to produce the catalytic and regulatory subunits of PI3Kα, PI3Kβ, and PI3Kδ. In the case of PI3Kγ, no regulatory subunit was required for activity [32].

SAR studies began by changing R_1_ from methyl (J27) to ethyl (J32). The potency for PI3Kα was retained but selectivity against the other three isoforms increased by up to 8-fold (Supplementary scheme 1, Table 1). Increasing the size of R_1_ by substituting ethyl (J32) with isopropyl (J55), iso-butyl (J46) or benzyl (J30) caused a 6 to 49-fold drop in inhibitory activity for PI3Kα. Polar groups such as ester (J43) and nitrile (J47) at R_1_ did not increase potency. A switch of the sulfonyl group at X to a carbonyl (J45) did improve inhibitory activity for PI3Kβ, PI3Kγ and PI3Kδ, but not for PI3Kα, thus decreased selectivity for the latter. With respect to the terminal phenyl ring, deleting a methyl group at R_2_ (J37) or a nitro group at R_3_ (J40) resulted in greater than 3.6-fold and 1333-fold decreases in inhibitory activity for PI3Kα, respectively. However, a chloro (J101) substituent at R_2_ was able to maintain potency and isoform selectivity as long as a nitro group was also present at R_3_. The R_3_ nitro group turned out to be critical in this configuration, as substitutions by various moieties, including carboxylic acid (J100), amino (J44), amido (J125) and methyl sulfonyl (J41) groups all impaired potency. We discovered that a substitution at the R_4_ position next to the nitro group on the phenyl ring was beneficial and that the R_4_ position in general accepted larger substituents without major impact on potency. Amino (J124-I), glycino (J159) and trifluoroacetylamino (J124) groups at the R_4_ position increased inhibitory activity for PI3Kα by 7.5, 3.8 and 1.4-fold, respectively, when compared to J32. Other substitutions, such as fluoro (J120), hydroxyl (J129) and various amino groups (J157, J158, J161, J162, J163), did not impact the inhibitory activity for PI3Kα substantially. Even larger substitutions as well as oligopeptide adducts retained nanomolar potencies (e.g. J171).

**Figure 1:**
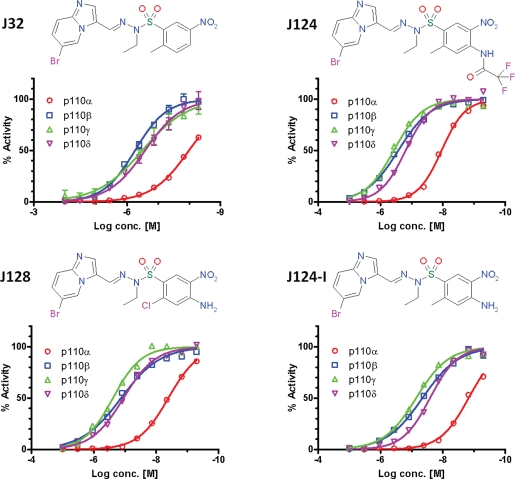
Biochemical activities of selected J-series compounds exemplify their PI3Kα selectivity and potency Inhibitor concentration is plotted against relative biochemical activity. Four PI3K isoforms were tested: alpha (red), beta (blue), gamma (green) and delta (purple). The structures of the compounds are shown above each panel.

In summary, seventy-four of the 121 compounds had a IC_50_ for PI3Kα below 1 μM, but of these, only 42 had IC_50_'s below 100 nM and 9 had IC_50_'s below 20 nM (examples in Fig. 1). The most specific of these (J171) had ~670 -fold selectivity against PI3Kβ, 60-fold against PI3Kγ, and 100-fold against PI3Kδ with a potency of 19 nM for PI3Kα (Table 1). More typical selectivity values were >20 (β), >30 (γ), and >10 (δ), respectively, with a potency for PI3Kα at 10 nM (e.g. J124). The compounds were also tested for their capacity to inhibit two common mutant forms of PI3Kα – E545K and H1047R [2, 33]. None of the compounds discriminated between the wild-type (wt) and mutant forms of the enzyme (data not shown). Finally, we also tested one of the compounds (J124, chosen for reasons discussed later) against a panel of 273 kinases. This compound proved to be moderately selective, inhibiting 22 of 273 tested kinases, but not mTOR, with an IC_50_ of 11 nM or lower (Supplementary Table 1).

**Table 1:** Structure-activity relationship table of representative J-series compounds. Representative compounds along with enzymatic potencies are listed.

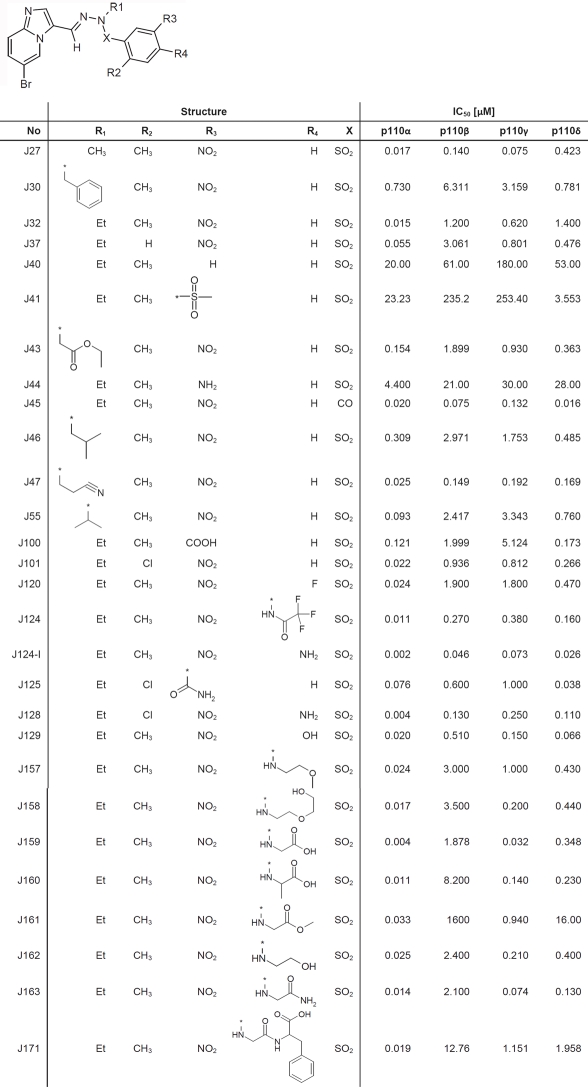

### J-series compounds inhibit the proliferation of cancer cells with and without mutant PI3Kα

**Figure 2:**
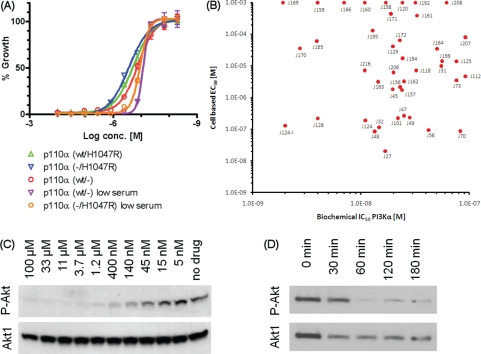
Cellular activity of J-series compounds (a), Efficacy of J124 in parental and isogenic HCT116 lines harboring wild-type or mutated *PIK3CA* alleles. (b), Activity matrix of cellular versus biochemical potency. Only J-series compounds with IC_50_ below 100 nM are shown. Compounds with no apparent cellular activity were assigned the default EC_50_ value of 1 mM. (c), Western blots demonstrating inhibitory effect of J124-I compound on phosphorylation of downstream effector Akt in HCT116 cells. (d), Intra-tumor P-Akt levels in HCT116 xenografts are reduced up to 3 hours post J124-I IP injection.

All 42 compounds with a PI3Kα IC_50_ below 100 nM were assayed for their ability to inhibit the growth of HCT116 cells in culture. These cells were derived from a human colorectal cancer containing one mutant (H1047R) and one normal allele of the *PIK3CA* gene. Paired isogenic lines in which one of the two alleles was disrupted through homologous recombination have been generated [8] and were also tested. We found that the 42 compounds inhibited cell growth to varying extents, but none of them inhibited the growth of the cells with a mutant *PIK3CA* allele more than their isogenic counterparts with only a normal allele (example in Fig. 2A). It has previously been demonstrated that the *PIK3CA* mutations allow cells to proliferate in growth medium containing limiting concentrations of growth factors [2]. Cells with both genotypes were more sensitive to the compounds when grown under conditions where growth factors were limiting, but these conditions did not provide specificity for the cells with a mutant PIK3CA allele (Fig. 2A)

To identify the most promising drug leads for in vivo evaluation, a matrix of cellular and biochemical potency of the 42 compounds with nanomolar IC_50_'s was constructed (Fig. 2B). We searched for potent compounds that inhibited cell growth at concentrations consistent with their ability to inhibit PI3Kα enzymatic activity. None of the compounds inhibited growth at concentrations less than their biochemical K_i_. Compounds that did not inhibit cell growth even at concentrations much greater than their K_i_'s were assumed to be cell impenetrant or inactive in an intracellular environment. Four compounds (J32, J124, J124-I, and J128) with biochemical and cellular activities which we considered optimal were chosen for further analysis.

To determine whether these compounds inhibited the pathway regulated by PI3Kα, we evaluated the phosphorylation of Akt1 and Akt2 in HCT116 cells following exposure to the compounds for 6 hours. Previous studies have demonstrated that the Akt1 and Akt2 proteins are reliable indicators of PI3Kα pathway activity [8, 28]. As assessed by western blot, the four compounds all inhibited phosphorylation of Akt1 and Akt2 when used at concentrations that inhibited cell growth (example in Fig. 2C).

### J-series compounds are potent and selective inhibitors of metastatic disease

We next tested these compounds in vivo. Through dose escalation studies, we found that the compounds were tolerated at concentrations up to 150 mg/kg when administered intraperitoneally daily for three weeks. Two of the compounds (J32, J124-I) were evaluated for their ability to inhibit the growth of subcutaneous HCT116 xenograft tumors in nude mice. Only a minor anti-tumor activity was noted (Supplementary Fig. 1), even though the compound inhibited the phosphorylation of Akt1/2 in the growing tumors (Fig. 2D).

**Figure 3:**
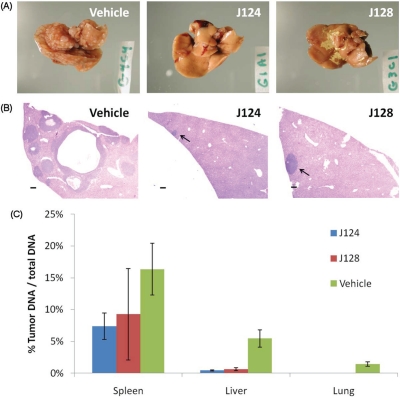
J124 and J128 have anti-metastatic efficacy in a metastasis model (a), Livers of mice treated with J124 and J128 as opposed to vehicle alone show distinctive difference in the number of tumor nodules. (b), Representative liver H&E sections of treated and untreated animals underscore differential liver metastasis load. Arrows indicate tumor lesions, bar length 200 μm. (c), Relative amount of tumor DNA in organs of treated and untreated animals. LINE-qPCR confirmed the reduced metastatic burden in livers of animals treated with J124 or J128.

To test the compounds in a context more relevant to the proposed tumorigenic role of PI3Kα, we evaluated their ability to inhibit the development of metastases from tumors injected into the spleen. Such injections give rise to large, primary intrasplenic tumors and multiple metastatic lesions in the liver, as well as a few tumor nodules in the lungs. The tumor-bearing animals were treated daily by intraperitoneal injections of J124 or J128 at 150 mg/kg starting three days after tumor implantation. Metastatic burdens were assessed through histopathology analysis three weeks later. All mice had large intrasplenic tumors, but the mice injected with J124 or J128 had few, if any, metastatic foci in their livers compared to animals injected with the vehicle alone (Fig. 3A). Sections of the liver and lungs revealed multiple tumor foci in control mice but not in mice treated with J124 or J128 (Fig. 3B).

To quantitatively measure metastatic tumor burdens in these mice, we performed real-time PCR using human-specific primers [34]. The spleens of the mice treated with J124 or J128 showed a modest reduction in primary tumor burdens (~50%) compared to the mice treated with only the vehicle (Fig. 3C). In contrast, the metastatic liver burden was reduced by 13-fold and 9-fold, respectively, in the mice treated with J124 and J128. There were a few metastatic lesions in the lungs of the control mice but no lesions in the lungs of the treated mice.

## DISCUSSION

We describe above a class of inhibitors that are highly specific for the alpha isoform of PI3K enzymes. Most published cell biologic and physiologic studies employing PI3Kα inhibitors have used either wortmannin or LY290042. These classic compounds have proven useful but are non-specific with respect to PI3K isoforms. The compounds we have synthesized should prove superior for such research purposes and will be made freely available upon request. Furthermore, the detailed evaluation of structure activity relationships provides leads for further optimization of this and related classes of compounds. Particularly intriguing was the discovery that the R_4_ position accepted very large substituents, even peptides, without major impact on potency. This finding sets the stage for the development of bi-functional compounds that may have uniquely specific activities.

Our studies also provide strong pharmacologic evidence for the role of PI3Kα in metastasis. Apart from the biologic implications of this finding, it has important practical ramifications. All anti-cancer compounds, including those targeting PI3Kα, are tested in pre-clinical models prior to their introduction into Phase I clinical trials. In general, therapeutic efficacy against a well-chosen tumor cell line is required to move forward. For example, PI3Kα inhibitors might be tested against xenografts of human cancer cell lines which have mutations of *PIK3CA*, for obvious reasons. Our results show that it is not only the choice of cell line that is important for pre-clinical testing, but also the choice of the in vivo model in which the testing is performed. J124 and J128 have little activity against subcutaneous tumors, or even against intra-abdominal primary tumors, but could potently inhibit the development of metastases. These results suggest that testing of novel compounds against tumors grown subcutaneously in immunocompromised mice could be misleading, stimulating researchers to discard drugs that might be useful in the clinic.

The pharmacologic data provided in this study are in perfect accord with prior genetic data. In particular, homologous recombination-mediated disruption of a mutant *PIK3CA* allele did not inhibit the growth of primary tumors but did inhibit metastatic growth [8]. No drug could possibly inhibit the mutant PI3Kα isoform as completely, permanently, and specifically as a genetic knock-out. We would thus argue that any drug that is designed to inhibit this enzyme should yield similar results in vivo, exhibiting minor effects on primary tumors and major effects on metastases. And conversely, drugs that inhibit the growth of primary tumors grown subcutaneously *must* be doing so by off-target effects. We believe that most of the drugs now being considered or used for clinical trials have not met this straight-forward expectation. On a positive note, attention to this expectation could lead to an improved selection of drugs for future clinical trials, thereby expediting the drug development process.

## MATERIALS AND METHODS

### Chemical compound synthesis

The full synthetic scheme and methods are described in Supplementary Scheme 1 and Supplementary Methods.

### PI3K expression and purification

cDNA clones of *PIK3CA*, *PIK3CB* and *PIK3CG* were obtained from Origene (Rockville, MD, USA). *PIK3CD* was generously provided by Novartis Pharmaceuticals. The cDNA clones were sub-cloned into pFastBac His-Tag (Invitrogen, Carlsbad, CA, USA) and baculovirus clones were generated by the Bac-to-Bac expression system (Invitrogen). p110α, p110β and p110δ were co-expressed in Sf9 insect cells (Invitrogen) with a nSH2-iSH2 fragment of the regulatory subunit p85 comprising AA residues 322 to 600 [4]. p110γ was expressed without a regulatory subunit. Sf9 cells were lysed in a buffer containing 50 mM sodium phosphate, pH 8.0, 400 mM NaCl, 5% glycerol, 1% Triton X-100, 10 mM 2-mercaptoethanol, 1 mM orthovanadate, 10 mM imidazole, and sonicated on ice for 1 minute. Proteins were purified by adding Ni-NTA Superflow beads (Qiagen, Valencia, CA, USA) to the lysate and subsequent rolling for 30 min at 4°C. Beads were washed twice with 50 mM phosphate buffer, pH 8.0, 0.5 M NaCl, 50 mM Imidazole, 2 mM DTT and eluted in the same buffer but with 200 mM imidazole. Purified PI3K's were mixed 1:1 with glycerol and stored at -20°C.

### Biochemical PI3K inhibition assay

J-series inhibitors were dissolved, serial diluted in DMSO and deposited into 96 well plates at a final DMSO concentration of 1%. We used a novel assay to determine their potency and selectivity. In brief, micelles of 2.5 μg L-a-Phosphatidylinositol and 2.5 μg 1,2-Diacyl-sn-glycero-3-phospho-L-serine were formed by sonication, mixed with the individual PI3K isoforms and added to the drug dilutions in a reaction buffer containing 10 mM HEPES, 25 mM NaCl, 0.125 μg/μl BSA, and 2 mM BME (final concentrations). Reactions were timed after the addition of 10 μM ATP with 40 μCi/ml γ-32P-ATP (PerkinElmer, Waltham, MA, USA) and 2 mM MgCl_2_ (all final concentrations). The reactions were terminated after two hours by the addition of 2 N HCl and the lipids extracted with 1:1 MetOH/Chloroform. Radio-labeled phosphatidyl-inositol-3-phosphate in the extracted organic fraction was quantified after the addition of Microscint O (PerkinElmer) using a TopCount 96 well plate scintillation counter (PerkinElmer). IC_50_ concentrations and inhibition curves were calculated using Prism 5 (GraphPad Software, La Jolla, CA).

### Cell-based inhibition assays

The colorectal cancer cell line HCT116 was obtained from ATCC and the generation of somatic knock-out derivatives with PI3Kα wt or H1047R genotypes has been described [8]. Cells were seeded in McCoy's 5A medium (Invitrogen)supplemented with 10% Fetal Bovine Serum (FBS, HyClone, Logan, UT, USA)at a density of 4000 cells per well in 96-well tissue culture plates (Corning, Corning, NY, USA). Once the cells had adhered to the plates (~5 hours after seeding), J-series inhibitors were added. All control and assay wells were adjusted to a final DMSO concentration of 1% and incubated for 48 hours. The media was removed and the adherent cells lysed by incubation for 1 hour at 37°C with 100 μl 10 mM Tris-HCl, pH 8.8, 0.5% Igepal (Sigma-Aldrich, St. Louis, MO, USA), 25 μg/ml Proteinase K (Invitrogen), and 0.05% SYBR green (Applied Biosystems, Carlsbad, CA, USA). DNA was measured with a FLUOstar Galaxy 96-well plate reader (BMG Labtech, Cary, NC, USA), and cell numbers were approximated by DNA content.

### P-Akt western analysis

Phopspho-Akt levels were assayed in HCT116 cells. 1x10^6^ cells/well were seeded in six-well plates and serum starved for 2 hours in McCoy's 5A medium. Drugs were pre-mixed with complete medium containing 10% FBS and incubated with the cells for 6 hours. Cells were lysed on ice with 100 mM Tris-HCl, pH 7.0, 10% 2-mercaptoethanol, 4% SDS, 20% glycerol, 0.05% Bromophenol blue, 20 μl 1 mM NaF, 1 mM Na_3_VO_4_, and Complete Protease Inhibitor Cocktail Tablet (Roche, Nutley, NJ, USA) without EDTA and sonicated for 30 seconds. Phospho-Akt was assayed with Phospho-Akt (Ser473) antibody (#9271, Cell Signaling Technology, Danvers, MA, USA). Subsequently the membrane was stripped and incubated with Akt1 (D-17) antibody (sc-7126, Santa Cruz Biotechnology,Santa Cruz, CA, USA)) for loading control.

### Intraspleenic tumor model

Intraspleenic-injected HCT116 liver and lung metastases in NOD.Cg-Prkdcscid Il2rgtm1Wjl/SzJ mice (Jackson Laboratory, Bar Harbor, ME, USA) were generated as described in Erikson *et. al.* [28]. J-series compounds were solubilized in 1:1 DMSO/Cremophor EL (Sigma-Aldrich) and diluted 2.3-fold in water to a final concentration of 7.5 mg/ml. 400 μl of this formulation (150 mg/kg) was injected intraperitoneally (IP) once a day for three weeks, beginning the third day post-surgery. Mice were sacrificed after the treatment and the liver, lungs, and spleen sampled. DNA was extracted from organs with the DNeasy Blood & Tissue Kit (Qiagen). The qPCR assay used for quantification of human DNA in mouse organs has been described [34]. All mouse protocols were designed in accordance with the NIH Guide for the Care and Use of Laboratory Animals and approved by the Institutional Animal Care and Use Committee.

### Xenograft tumor model

Tumor xenografts were established by injecting 5x10^6^ HCT116 cells subcutaneously into the right flanks of female nude mice. Tumor volume was measured twice weekly using electronic calipers and calculated as length x width^2^ x 0.5. After tumors reached an average volume of 50 mm^3^, animals were treated daily with drugs at 150 mg/kg via IP injection for three weeks. Tumor phospho-Akt levels were determined following harvest on the last day of the study. Xenografts were excised, snap frozen in liquid nitrogen and pulverized with a pestle. Homogenized tissue was re-suspended in lysis buffer (Cell Signaling Technology) and protein contents normalized by Bradford assay (Bio-Rad Laboratories, Hercules, CA, USA). Western blots were performed as described above.

## SUPPLEMENTARY MATERIALS



## References

[1] Engelman J.A., Luo J., Cantley L.C. (2006). The evolution of phosphatidylinositol 3-kinases as regulators of growth and metabolism. Nat. Rev. Genet..

[2] Samuels Y., Wang Z., Bardelli A., Silliman N., Ptak J., Szabo S., Yan H., Gazdar A., Powell S.M., Riggins G.J., Willson J.K., Markowitz S., Kinzler K.W., Vogelstein B., Velculescu V.E. (2004). High frequency of mutations of the PIK3CA gene in human cancers. Science.

[3] Vogt P.K., Kang S., Elsliger M.A., Gymnopoulos M. (2007). Cancer-specific mutations in phosphatidylinositol 3-kinase. Trends Biochem. Sci..

[4] Huang C.H., Mandelker D., Schmidt-Kittler O., Samuels Y., Velculescu V.E., Kinzler K.W., Vogelstein B., Gabelli S.B., Amzel L.M. (2007). The structure of a human p110alpha/p85alpha complex elucidates the effects of oncogenic PI3Kalpha mutations. Science.

[5] Isakoff S.J., Engelman J.A., Irie H.Y., Luo J., Brachmann S.M., Pearline R.V., Cantley L.C., Brugge J.S. (2005). Breast cancer-associated PIK3CA mutations are oncogenic in mammary epithelial cells. Cancer Res..

[6] Kang S., Bader A.G., Vogt P.K. (2005). Phosphatidylinositol 3-kinase mutations identified in human cancer are oncogenic. Proc. Natl. Acad. Sci. USA.

[7] Mandelker D., Gabelli S.B., Schmidt-Kittler O., Zhu J., Cheong I., Huang C.H., Kinzler K.W., Vogelstein B., Amzel L.M. (2009). A frequent kinase domain mutation that changes the interaction between PI3Kalpha and the membrane. Proc. Natl. Acad. Sci. USA.

[8] Samuels Y., Diaz L.A., Schmidt-Kittler O., Cummins J.M., Delong L., Cheong I., Rago C., Huso D.L., Lengauer C., Kinzler K.W., Vogelstein B., Velculescu V.E. (2005). Mutant PIK3CA promotes cell growth and invasion of human cancer cells. Cancer Cell.

[9] Vanhaesebroeck B., Waterfield M.D. (1999). Signaling by distinct classes of phosphoinositide 3-kinases. Exp. Cell Res..

[10] Chen J.S., Zhou L.J., Entin-Meer M., Yang X., Donker M., Knight Z.A., Weiss W., Shokat K.M., Haas-Kogan D., Stokoe D. (2008). Characterization of structurally distinct, isoform-selective phosphoinositide 3'-kinase inhibitors in combination with radiation in the treatment of glioblastoma. Mol. Cancer Ther..

[11] Fan Q.W., Cheng C.K., Nicolaides T.P., Hackett C.S., Knight Z.A., Shokat K.M., Weiss W.A. (2007). A dual phosphoinositide-3-kinase alpha/mTOR inhibitor cooperates with blockade of epidermal growth factor receptor in PTEN-mutant glioma. Cancer Res..

[12] Foster P. (2007). Potentiating the antitumor effects of chemotherapy with the selective PI3K inhibitor XL147. 19th AACR-NCI-EORTC Meeting.

[13] Garlich J.R., De P., Dey N., Su J.D., Peng X., Miller A., Murali R., Lu Y., Mills G.B., Kundra V., Shu H.K., Peng Q., Durden D.L. (2008). A vascular targeted pan phosphoinositide 3-kinase inhibitor prodrug, SF1126, with antitumor and antiangiogenic activity. Cancer Res..

[14] Hayakawa M., Kawaguchi K., Kaizawa H., Koizumi T., Ohishi T., Yamano M., Okada M., Ohta M., Tsukamoto S., Raynaud F.I., Parker P., Workman P., Waterfield M.D. (2007). Synthesis and biological evaluation of sulfonylhydrazone-substituted imidazo [1,2-a]pyridines as novel PI3 kinase p110alpha inhibitors. Bioorg. Med. Chem..

[15] Howes A.L., Chiang G.G., Lang E.S., Ho C.B., Powis G., Vuori K., Abraham R.T. (2007). The phosphatidylinositol 3-kinase inhibitor, PX-866, is a potent inhibitor of cancer cell motility and growth in three-dimensional cultures. Mol. Cancer Ther..

[16] Knight Z.A., Chiang G.G., Alaimo P.J., Kenski D.M., Ho C.B., Coan K., Abraham R.T., Shokat K.M. (2004). Isoform-specific phosphoinositide 3-kinase inhibitors from an arylmorpholine scaffold. Bioorg. Med. Chem..

[17] Knight Z.A., Gonzalez B., Feldman M.E., Zunder E.R., Goldenberg D.D., Williams O., Loewith R., Stokoe D., Balla A., Toth B., Balla T., Weiss W.A., Williams R.L., Shokat K.M. (2006). A pharmacological map of the PI3-K family defines a role for p110alpha in insulin signaling. Cell.

[18] Norman B.H., Shih C., Toth J.E., Ray J.E., Dodge J.A., Johnson D.W., Rutherford P.G., Schultz R.M., Worzalla J.F., Vlahos C.J. (1996). Studies on the mechanism of phosphatidylinositol 3-kinase inhibition by wortmannin and related analogs. J. Med. Chem..

[19] Raynaud F.I. (2007). Pharmacologic characterization of a potent inhibitor of class I phosphatidylinositide 3-kinases. Cancer Res..

[20] Raynaud F.I. (2009). Biological properties of potent inhibitors of class I phosphatidylinositide 3-kinases: from PI-103 through PI-540, PI-620 to the oral agent GDC-0941. Mol. Cancer Ther..

[21] Shapiro G., Edelman G., Calvo E., Aggarwal S., Laird A. (2007). Targetting aberrant PI3K pathway signaling with XL147, a potent selective, and orally bioavailable PI3K inhibitor. 19th AACR-NCI-EORTC Meeting.

[22] Ihle N.T., Williams R., Chow S., Chew W., Berggren M.I., Paine-Murrieta G., Minion D.J., Halter R.J., Wipf P., Abraham R., Kirkpatrick L., Powis G. (2004). Molecular pharmacology and antitumor activity of PX-866, a novel inhibitor of phosphoinositide-3-kinase signaling. Mol. Cancer Ther..

[23] Maira S.M. (2008). Identification and characterization of NVP-BEZ235, a new orally available dual phosphatidylinositol 3-kinase/mammalian target of rapamycin inhibitor with potent in vivo antitumor activity. Mol. Cancer. Ther..

[24] Molckovsky A., Siu L.L. (2008). First-in-class, first-in-human phase I results of targeted agents: highlights of the 2008 American society of clinical oncology meeting. J. Hematol. Oncol..

[25] Kong D., Yamori T. (2009). Advances in development of phosphatidylinositol 3-kinase inhibitors. Curr. Med. Chem..

[26] Edgar K.A., Wallin J.J., Berry M., Lee L.B., Prior W.W., Sampath D., Friedman L.S., Belvin M. (2010). Isoform-specific phosphoinositide 3-kinase inhibitors exert distinct effects in solid tumors. Cancer Res..

[27] Maira M., Menezes D., Pecchi S. (2010). Biological characterization of NVP-BKM120, a novel inhibitor of phosphoinosotide 3-kinase in Phase I/II clinical trials. 101st American Association for Cancer Research Congress.

[28] Ericson K., Gan C., Cheong I., Rago C., Samuels Y., Velculescu V.E., Kinzler K.W., Huso D.L., Vogelstein B., Papadopoulos N. (2009). Genetic inactivation of AKT1, AKT2, and PDPK1 in human colorectal cancer cells clarifies their roles in tumor growth regulation. Proc. Natl. Acad. Sci. USA.

[29] Rychahou P.G., Kang J., Gulhati P., Doan H.Q., Chen L.A., Xiao S.Y., Chung D.H., Evers B.M. (2008). Akt2 overexpression plays a critical role in the establishment of colorectal cancer metastasis. Proc. Natl. Acad. Sci. USA.

[30] Chen J.S., Wang Q., Fu X.H., Huang X.H., Chen X.L., Cao L.Q., Chen L.Z., Tan H.X., Li W., Bi J., Zhang L.J. (2009). Involvement of PI3K/PTEN/AKT/mTOR pathway in invasion and metastasis in hepatocellular carcinoma: Association with MMP-9. Hepatol. Res..

[31] Davies M.A., Stemke-Hale K., Lin E., Tellez C., Deng W., Gopal Y.N., Woodman S.E., Calderone T.C., Ju Z., Lazar A.J., Prieto V.G., Aldape K., Mills G.B., Gershenwald J.E. (2009). Integrated Molecular and Clinical Analysis of AKT Activation in Metastatic Melanoma. Clin. Cancer Res..

[32] Voigt P., Dorner M.B., Schaefer M. (2006). Characterization of p87PIKAP, a novel regulatory subunit of phosphoinositide 3-kinase gamma that is highly expressed in heart and interacts with PDE3B. J. Biol. Chem..

[33] Zhao L., Vogt P.K. (2008). Class I PI3K in oncogenic cellular transformation. Oncogene.

[34] Rago C., Huso D.L., Diehl F., Karim B., Liu G., Papadopoulos N., Samuels Y., Velculescu V.E., Vogelstein B., Kinzler K.W., Diaz L.A. (2007). Serial assessment of human tumor burdens in mice by the analysis of circulating DNA. Cancer Res..

