# Supplementary material for: PI3Kα Inhibitors That Inhibit Metastasis
Source: Oncotarget. 2010 Sep 11;1(5):339–48. doi: 10.18632/oncotarget.166 (PMC3004370; doi:10.18632/oncotarget.166)
Supplement: Supplementary file 1 [file oncotarget-01-339-s001.doc]

**PI3Kα inhibitors that inhibit metastasis**

Supplementary information

**Supplementary Scheme 1. Synthetic scheme of imidazopyridine-based J-series compounds.** General synthetic scheme for J-series compounds is shown here.

**Supplementary Figure 1. Effect of J124-I on HCT116 xenograft tumors.** Nude mice carrying subcutaneous HCT116 tumors were administered either vehicle or J124-I daily by intraparanual injections at 150 mg/kg for three weeks. Tumor volumes were recorded. Means and standard errors of five animals per experimental arm are illustrated.

**Supplementary Table 1. Millipore KinaseProfiler excerpt**

| **J124 @ 0.011 µM** | **% of control** |
| --- | --- |
| Haspin | -1 |
| Flt3(D835Y) | 3 |
| CLK2 | 5 |
| Flt3 | 10 |
| PTK5 | 11 |
| TrkA | 11 |
| MELK | 23 |
| ACK1 | 30 |
| Mer | 33 |
| PKCθ | 35 |
| LIMK1 | 36 |
| CDK5/p35 | 37 |
| Yes | 40 |
| IRAK1 | 42 |
| NEK2 | 42 |
| EGFR(T790M,L858R) | 46 |
| EphB1 | 46 |
| TBK1 | 46 |
| cSRC | 48 |
| IRAK4 | 48 |
| Src(1-530) | 48 |
| Ret | 49 |

**Supplementary Methods**

*Materials:*

All chemicals and solvents were purchased from Sigma-Aldrich (St. Louis, MO), Matrix Scientific (Columbia, SC), and Fisher Scientific (Pittsburgh, PA). ^1^H NMR spectra were recorded on Bruker 400MHz spectrometer (Bruker,Billerica, MA ), reported in parts per million on the δ scale, and are referenced from the residual protium in the NMR solvent (CDCl_3_: δ 7.24 (CHCl_3_). Data is reported as follows: chemical shift [multiplicity (s = singlet, d = doublet, t = triplet, q = quartet, m = multiplet), coupling constant(s) in Hertz, integration]. HRMS spectra were recorded on VG analytical VG-70S magnetic sector mass spectrometer (VG Analytical, UK). Reactions were monitored by TLC using EMD Silica gel 60 F_254_. (Fisher Scientific, Pittsburg, PA ) Chromatographic purification was performed as flash chromatography on SAI silica 63-200µM, 60 Å. (SAI, Altlanta,GA )

*General procedure for the preparation of 3:*

Please refer to Mandelker, D. *et al.* and Hayakawa, M*. et al.* for synthesis of 2 and 3 [1, 2].

*General procedure for the preparation of 5:*

4 (1mmol) was dissolved in chloroform (5ml). To the solution, chlorosulfonic acid (3mmol) is slowly added. Then the mixture was refluxed for 2hrs. After it’s cooled to rt, the solution was slowly poured to ice water with stirring. Compound 5 was extracted with chloroform, dried over MgSO_4_ and concentrated under vacuum.

*General procedure for the preparation of 6:*

To a suspension of 3 (1mmol) in 2mL DCM on ice was added Et_3_N (1.5mmol). Then 5 (1.2mmol) in 2mL DCM was added to the mixture. After it’s stirred at rt overnight, the mixture was concentrated and re-suspended in 15mL H_2_O. Product was extracted with 15mL DCM three times. The organic layer was combined, dried over NaSO_4_, concentrated and further purified with silica gel column to give compound 6

*J124*

(E)-N-(4-(2-((6-bromoimidazo[1,2-a]pyridin-3-yl)methylene)-1-ethylhydrazinylsulfonyl)-5-methyl-2-nitrophenyl)-2,2,2-trifluoroacetamide. mp: 228-230 °C; ^1^H-NMR (400MHz, CDCl_3_): δ 11.65(bs,1H), 9.11(s, 1H), 9.07(dd, J=0.8, 1.6Hz, 1H), 8.82(s, 1H), 8.16(s, 1H), 7.93(s, 1H), 7.62(dd, J=0.8, 9.6Hz, 1H), 7.43(dd, J=1.6, 9.6Hz, 1H), 4.06(q, J=7.2Hz, 2H), 2.75(s, 3H), 1.42(t, J=7.2Hz, 3H); HRMS (m/z): [M]^+^ calcd. for C_19_H_16_BrF_3_N_6_O_5_S, 576.0038/578.0018; obsd, 576.0044/578.0019

*J124-I*

(E)-4-amino-N'-((6-bromoimidazo[1,2-a]pyridin-3-yl)methylene)-N-ethyl-2-methyl-5-nitrobenzenesulfonohydrazide. mp: 247-248 °C; ^1^H-NMR (400MHz, MeOD): δ 9.13(dd, J=0.8, 2.0Hz,1H), 8.91(s,1H), 8.17(s,1H), 7.89(s,1H), 7.55(dd, J=0.8, 9.2Hz,1H), 7.46(dd, J=2.0, 9.2Hz,1H), 6.85(s,1H), 4.07(q, J=7.2Hz,2H), 2.48(s,3H), 1.37(t, J=7.2Hz,3H); HRMS (m/z): [M]^+^ calcd. for C_17_H_17_BrN_6_O_4_S, 480.0215/482.0195.; obsd, 480.0215/482.0189

*J128*

(E)-4-amino-N'-((6-bromoimidazo[1,2-a]pyridin-3-yl)methylene)-2-chloro-N-ethyl-5-nitrobenzenesulfonohydrazide. mp: 239-242 °C; ^1^H-NMR (400MHz, CDCl_3_:MeOD=98:2 v/v): δ 9.03(d, J=2.0Hz, 1H), 8.94(s, 1H), 7.91(s, 1H), 7.72(s, 1H), 7.41(d, J=9.2Hz, 1H), 7.30(dd, J=2.0, 9.2Hz, 1H), 6.93(s, 1H), 4.00(q, J=7.2Hz, 2H), 1.27(t, J=7.2Hz, 3H); HRMS (m/z): [M]^+^ calcd. for C_16_H_14_BrClN_6_O_4_S, 499.9669/501.9649; obsd, 499.9674/501.9645.

*References*

1. Hayakawa, M., K. Kawaguchi, H. Kaizawa, T. Koizumi, T. Ohishi, M. Yamano, M. Okada, M. Ohta, S. Tsukamoto, F.I. Raynaud, P. Parker, P. Workman, and M.D. Waterfield, Synthesis and biological evaluation of sulfonylhydrazone-substituted imidazo[1,2-a]pyridines as novel PI3 kinase p110alpha inhibitors*.* Bioorg. Med. Chem., 2007. 15(17): p. 5837-44.

2. Mandelker, D., S.B. Gabelli, O. Schmidt-Kittler, J. Zhu, I. Cheong, C.H. Huang, K.W. Kinzler, B. Vogelstein, and L.M. Amzel, A frequent kinase domain mutation that changes the interaction between PI3Kalpha and the membrane*.* Proc. Natl. Acad. Sci. USA, 2009. 106(40): p. 16996-7001.
